# Prenatal MRI diagnosis and outcomes of abdominal or sacrococcygeal teratomas and parasitic fetuses

**DOI:** 10.3389/fped.2023.1181110

**Published:** 2023-09-06

**Authors:** Xu Li, Hui hui Lin, Ke fei Hu, Yun Peng

**Affiliations:** ^1^ Department of Radiology, Anhui Provincial Children’s Hospital, Hefei, China; ^2^ Department of Radiology, Beijing Children's Hospital, Capital Medical University, Beijing, China

**Keywords:** fetus, teratoma, parasitic fetus, magnetic resonance imaging, outcomes

## Abstract

**Objective:**

To investigate the MRI findings of fetal abdominal or sacrococcygeal teratomas and parasitic fetuses and analyze the outcomes on the basis of follow-up assessments.

**Methods:**

The MRI data of 60 cases of abdominal or sacrococcygeal masses were examined. The outcomes were followed up and compared with the prenatal MRI diagnoses.

**Results:**

The 60 cases included 52 cases of sacrococcygeal teratomas and eight cases of abdominal lesions. The common types of sacrococcygeal teratomas were type I (21/52, 40.4%) and type II (20/52, 38.5%); type III sacrococcygeal teratomas were rarer (8/52, 15.4%), while type IV tumors (3/52, 5.7%) were frequently complicated with hydronephrosis. Other complications included polyhydramnios in 22 cases, placental edema in six cases, and fetal hydronephrosis in three cases (all type IV). Seven of the eight parasitic fetuses were located in the abdominal cavity, and one was located in the sacrococcygeal region. Postnatal surgery was performed in 51 cases (51/60), including 44 with teratomas and seven with parasitic fetuses. In one case with hydronephrosis, peritoneal effusion, and subcutaneous edema, treatment was discontinued after birth (1/60). Fetal induction of labor was observed in eight cases (8/60). Prenatal ultrasound yielded incorrect or ambiguous diagnoses in 11 cases, while 51 cases showed a favorable course after surgery.

**Conclusions:**

MRI shows high accuracy in the diagnosis of fetal sacrococcygeal teratomas and parasitic fetuses. The prognosis in these cases is generally good. However, type IV sacrococcygeal teratomas are prone to fetal hydronephrosis and misdiagnosis and show a poorer prognosis.

## 1. Introduction

Fetal teratomas are the most common congenital tumors in the fetus (1). Fetal teratomas are mainly diagnosed by ultrasound (2, 3), which shows very high sensitivity (100%) and low false-positive (3.3%) rates in the prenatal identification of teratomas. The specificity rate (96.7%) and positive predictive value (83.3%) of ultrasound have also been reported to be high (4). The most frequent locations of these tumors are the sacrococcygeal region, the neck, and the oropharyngeal cavity, while less common locations included the brain, pericardium, mediastinum, abdomen, and testes (5). The classification system for sacrococcygeal teratomas was established by the Surgical Section of the American Academy of Pediatrics, and these lesions were categorized into four types: (I) entirely external with a small tumor component at the coccyx, (II) predominantly external with a smaller intrapelvic component, (III) predominantly intrapelvic with a smaller external component, and (IV) entirely intrapelvic or pre-sacral. Prenatal diagnosis can improve the perinatal management of these lesions, especially for teratomas that may benefit from fetal intervention (6). On the other hand, parasitic fetuses are rare malformations characterized by the presence of a parasitic twin within a more mature twin. The characteristics of parasitic fetuses include a mass that is enclosed within a distinct fibrous membrane, is partially or completely covered by normal skin, shows grossly recognizable anatomic structures, and is supplied by a relatively large blood vessel from the host fetus; most parasitic fetuses are located in the retroperitoneum and are cardiac and anencephalic (7). Less than 200 cases of parasitic fetus have been reported in the literature (6). Prenatal ultrasound can identify rudimentary organs indicating parasitic fetuses from early pregnancy. Detection of the fetal heartbeat facilitates differential diagnosis from teratomas, providing essential information for parental consulting and management (8).

Magnetic resonance imaging (MRI) provides a large field of view and allows arbitrary plane imaging with high soft-tissue resolution. MRI examinations are less affected by the week of conception and the amount of amniotic fluid, and the location and qualitative diagnosis of fetal abdominal and pelvic masses in MRI scans can provide additional information than that obtained with prenatal ultrasound. However, only a few reports with small sample sizes have described the use of fetal MRI for these diagnoses (9, 10). Therefore, the present study mainly aimed to evaluate the value of MRI in the diagnosis of fetal abdominal and sacrococcygeal teratomas or parasitic fetuses and to identify the possible prognostic factors and other factors affecting the prognosis through follow-up assessments. Through these findings, we hope that this study can further improve the effectiveness of perinatal consultation.

## 2. Material and methods

### 2.1. Clinical data

This retrospective analysis was performed using the data obtained for 60 pregnant women diagnosed with abdominal or sacrococcygeal masses by fetal MRI at our hospital from January 2013 to January 2022, including one case of twin pregnancy and 59 cases with a single fetus. The age of the pregnant women ranged from 22 to 38 years (mean, 27.5 ± 5.15 years), and their gestational age (calculated on the basis of the last menstruation date) ranged from 14 to 38 weeks (mean, 27.2 ± 4.65 weeks). All prenatal ultrasound diagnosis results were scanned and preserved in the hospital's picture archiving and communication system (PACS). Written informed consent was obtained from all pregnant women examined in the study or from their family members.

### 2.2. Scan parameters and scan range

A Philips Achieva 1.5 T MRI unit with a 16-channel abdominal coil was used for scanning. The scans were conducted by an MRI radiologist. The scans were performed with the pregnant women in a supine position or lying on the left side with their foot advanced and did not involve injection of sedatives and contrast agents or breath-holding. The scanning sequences included single-shot fast spin echo (SSFSE), balanced fast-field echo (BFFE), and T1-weighted imaging (T1WI). The scanning parameters were as follows: SSFSE, repetition time (TR) = 12,000.0–15,000.0 ms, excitation time (TE) = 120.0 ms, layer thickness = 4.0–6.0 mm, layer interval = 1.0–0 mm; BFFE, TR/TE = 3.7 ms/1.84 ms, layer thickness = 4.0–6.0 mm, layer interval = −3.0 to −2.0 mm; T1WI, TR/TE = 400 ms/15 ms, layer thickness = 5–7 mm, and fractional anisotropy (FA) = 15°. The field of view (FOV) for the scans was 310–350 mm × 310–350 mm, and the scans were performed with a 256 × 256 matrix.

The scope of abdominal and sacrococcygeal scanning included the use of SSFSE sequences to scan the transverse, coronal, and sagittal sections of the fetus's chest and abdomen, focusing on the size, shape, and signal characteristics of the mass, its relationship with adjacent organs, and the relationship between the sacrococcygeal lesion and vertebral lumen and vertebral body. The sagittal section of T1WI was used to observe the positional relationship between the lesion and the rectum. Routine SSFSE sequence scans of the fetal brain cross-section and BFFE scans of fetal heart cross-section were performed to identify any obvious brain structural malformations and cardiac macrovascular malformations.

### 2.3. Image analysis

Prenatal ultrasound results were known to the radiologist before scanning, and all fetal MRI images were carefully observed and diagnosed by two radiologists. In cases involving is disagreement, the final decision was made after discussions by the two radiologists.

## 3. Results

### 3.1. MRI diagnosis results in this group

**Figure 1:**
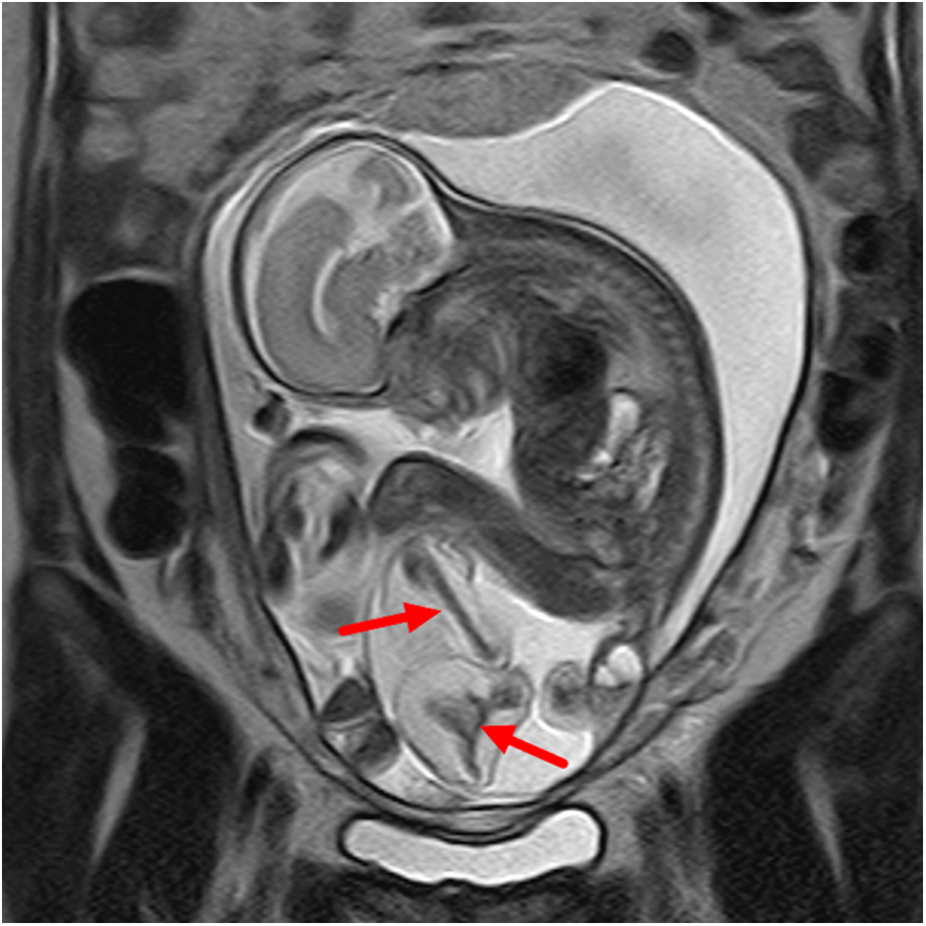
Sacrococcygeal parasitic fetus at 25 weeks of gestation. The sacrococcygeal mass has long limbs (red arrows).

**Figure 2:**
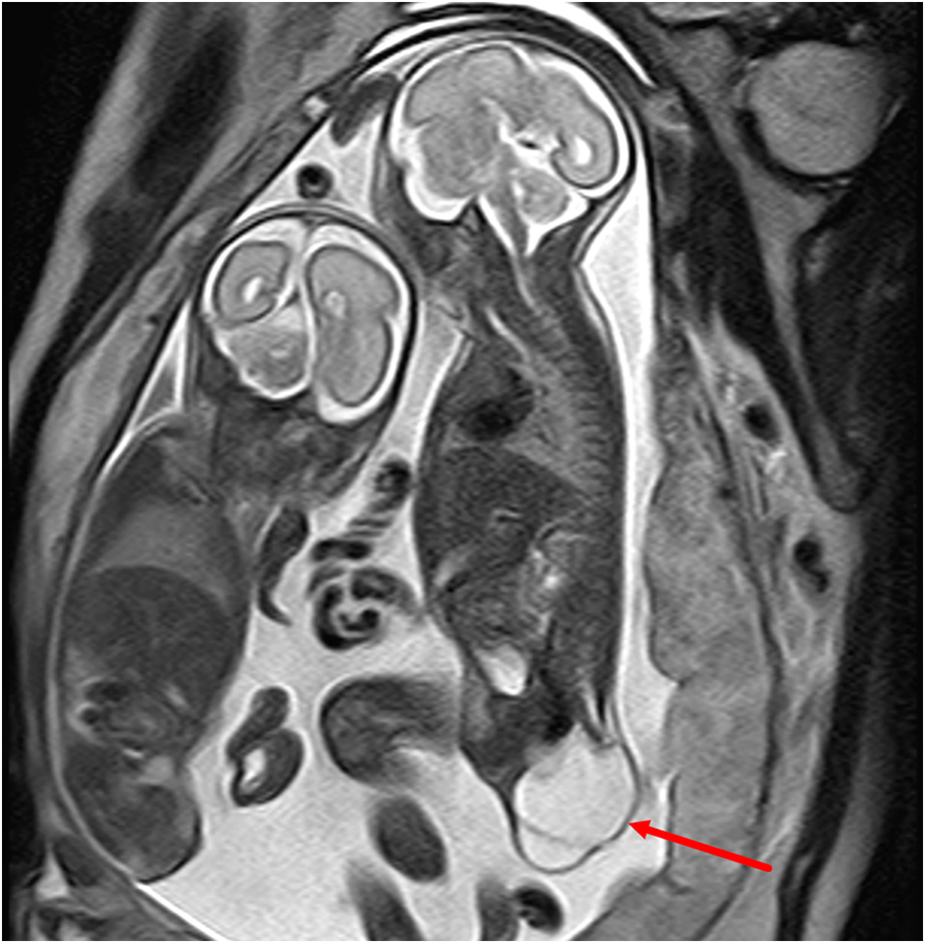
A type I sacrococcygeal teratoma (left) in a twin pregnancy at 24 weeks of gestation. A dominant mass is seen in the sacrococcygeal region of the fetus, which is dominated by cystic elements (red arrows).

**Figure 3:**
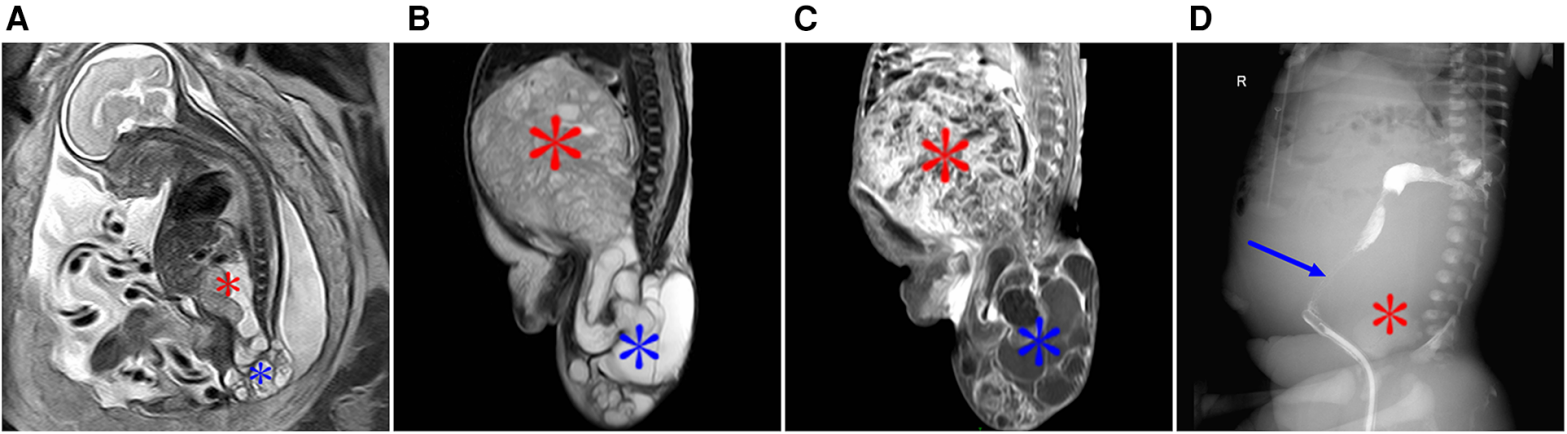
A type II sacrococcygeal teratoma at 25 weeks of gestation. (**A**) Mixed signals occupying both the front and the lower part of the sacrococcygeal region of the fetus. The lesions in the front of the sacrococcygeal region were mainly soft-tissue signals (red star), while the lesions in the lower part of the sacral tail mainly showed multiple cystic components (blue star). (**B**) After birth, the anterior sacral caudal mass on T2-weighted imaging showed iso-hyperintensity (red star), and the lesions below the sacral caudal mass were mainly cystic (blue star) and were significantly larger than those in the fetal period. (**C**) After enhanced scanning, the solid components of the lesion showed significant enhancement, and the cystic partial wall showed linear enhancement. (**D**) Lipiodol enema demonstrates a compressed anteriorly displaced rectum.

**Figure 4:**
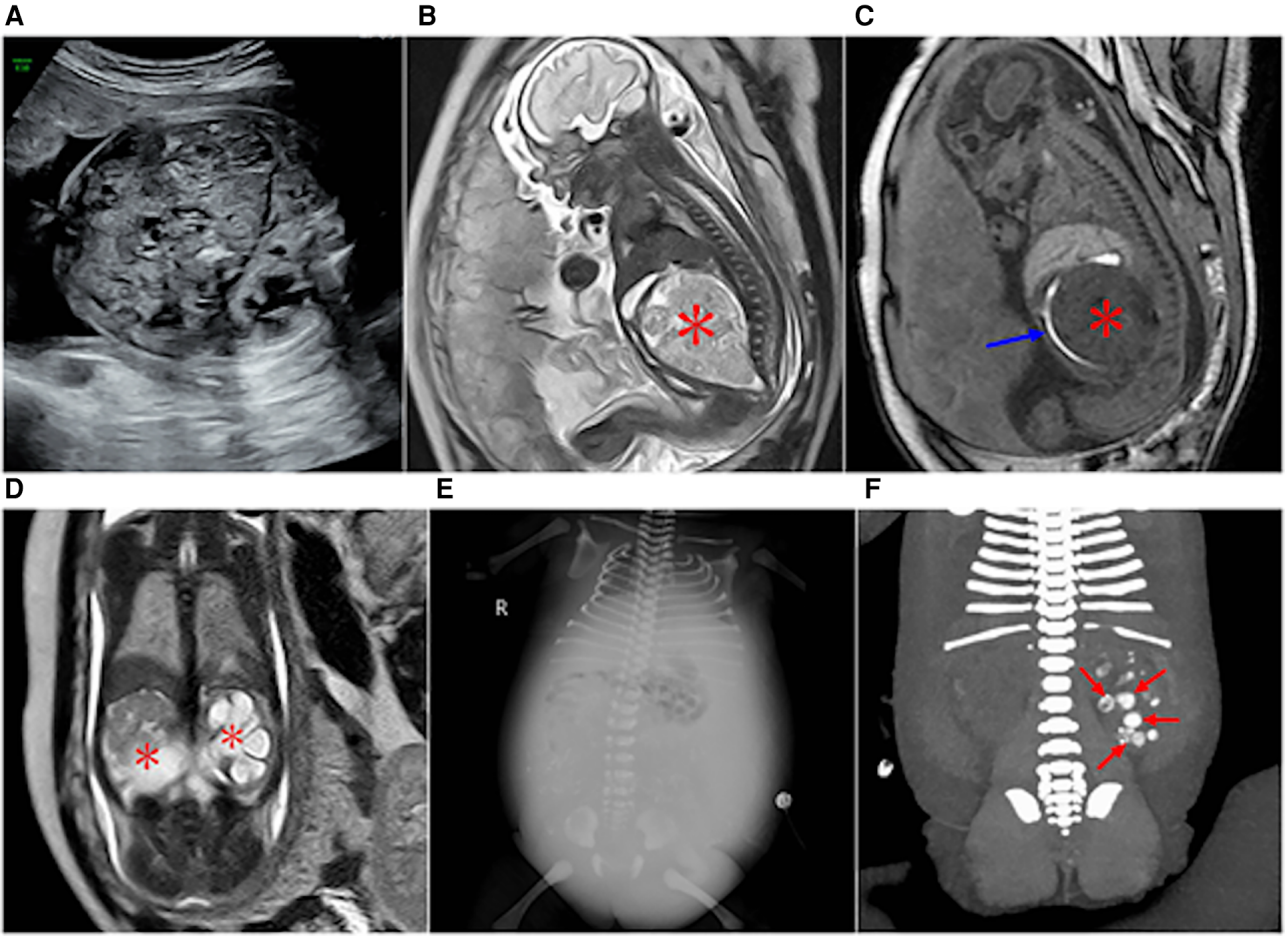
Type IV sacrococcygeal teratoma at 32 weeks of gestation. (**A**) Mixed echo with a clear boundary was found in the fetal abdominal cavity, and CDFI showed a strip-like blood flow signal in the echo. A small liquid dark area was found around the high echo. (**B**) A large isometric mass (asterisk) is seen anterior to the sacral coccyx. (**C**) T1WI shows iso-hypointensity as seen in the anterior sacrococcygeal lesion, and the rectal compression moved forward (blue arrow). (**D**) Significant hydronephrosis of both kidneys, which was obvious on the left side (asterisk). (**E**,**F**) Images obtained two days after the birth of the fetus in Figure 3. (**E**) Obvious abdominal wall and lower limb edema, reduced abdominal bowel gas accumulation, and abdominal fluid accumulation and scattered dot calcification density in the middle abdomen. (**F**) Coronal reconstruction on plain CT scans showing multiple nodular hyperintensities in the left kidney with possible calcification following necrotic hemorrhage (red arrow).

**Figure 5:**
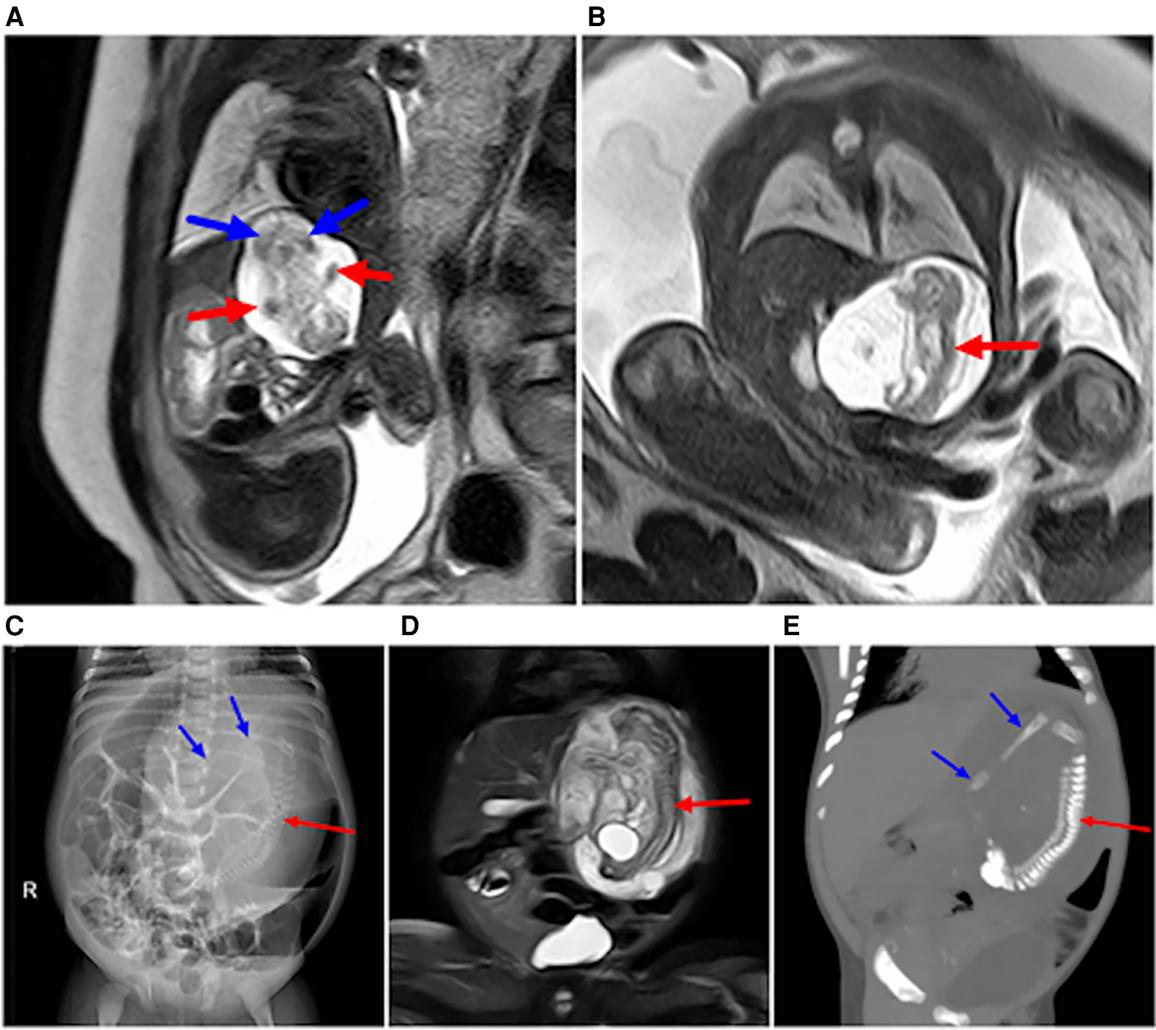
Image obtained at 31 weeks of gestation showing a parasitic fetus in the left upper abdomen. (**A**) Sagittal view of the fetus shows the left upper abdominal cystic mass, which contains a humanoid part of the limb structure, upper limb (red arrow), and lower limb (blue arrow). (**B**) Coronal view of the fetus showing a spinal structure within a left upper abdominal cystic lesion (red arrow). (**C**–**E**) Images obtained after birth of the fetus in Figure 6. (**C**) The left upper abdominal mass was larger than that of the fetus, and the dense mass occupying the left upper abdomen on plain abdominal film was visible, showing the complete spinal structure (red arrow) and part of the lower limb (blue arrow). (**D**) T2WI MRI images showing an enlarged left upper abdominal teratoma in comparison with fetal lesions, with a complete spinal pattern (red arrow). (**E**) 3D CT reconstruction shows the complete spinal structure of the parasitic fetus (red arrow) and lower limb (blue arrow).

**Figure 6:**
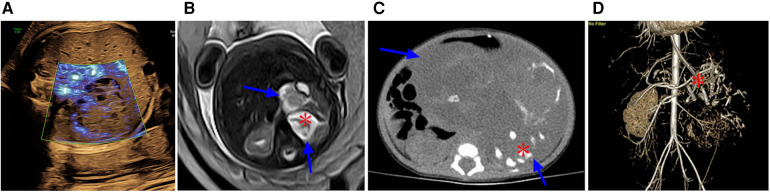
(**A**) At 29 weeks of gestation, a solid cystic mass with a clear boundary was observed in front of the fetal left kidney. Prenatal ultrasound indicated an adrenal solid cystic mass. (**B**) Image obtained at 29 weeks of gestation showing a parasitic fetus in the left upper abdomen. T2WI in the transverse view showed two interconnecting lesions in the left upper abdomen (blue arrow). The lesions in the rear were mainly cystic, and suspicious skeletal structures could be seen in the cyst (asterisk). (**C**,**D**) Images obtained six days after the birth of the fetus in image A and B. (**C**) Plain CT scan showed a significantly enlarged lesion with predominantly cystic components (blue arrows) and skeletal components (red asterisk) in the left upper abdomen; the lesion size was approximately four times larger than that at 29 weeks of gestation. (**D**) CT-enhanced 3D reconstruction with multiple long bone components in the left upper abdomen (red asterisk).

**Figure 7:**
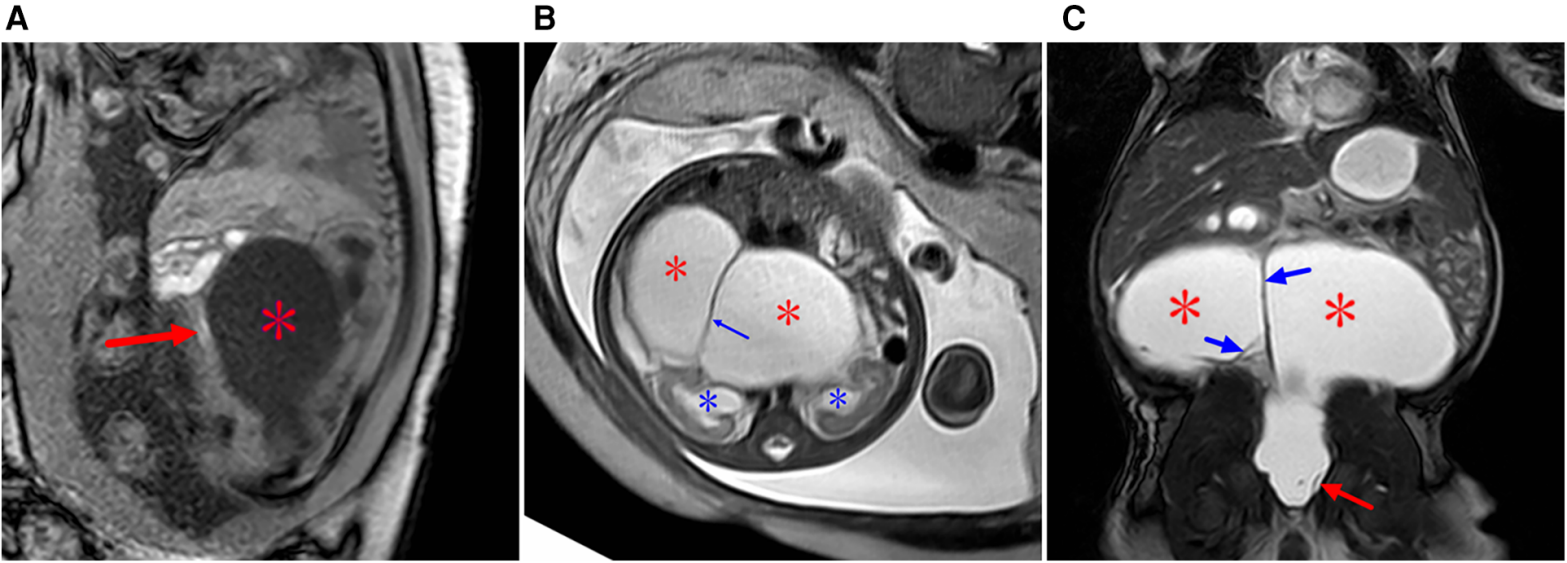
Type IV sacrococcygeal teratoma (misdiagnosed as cloacal malformation on fetal MRI) at 33 weeks of gestation. (**A**) On T1WI, the ellipsoidal low signal mass (red star) is seen in front of the sacral coccyx, and a strip of high signal (suspected rectum) is seen faintly in front of the lesion (red arrow). (**B**) The lesion is seen posteriorly in the abdominal pelvis, showing high signal intensity on T2WI, with linear separations (blue arrows) showing the left and right halves (red stars), and bilateral hydronephrosis of the fetus (blue asterisk). (**C**) On T2WI MRI after birth, the sacrococcygeal mass (red star) is seen with multiple separations (blue arrows) originating in the sacral caudal mass (red arrows).

**Figure 8:**
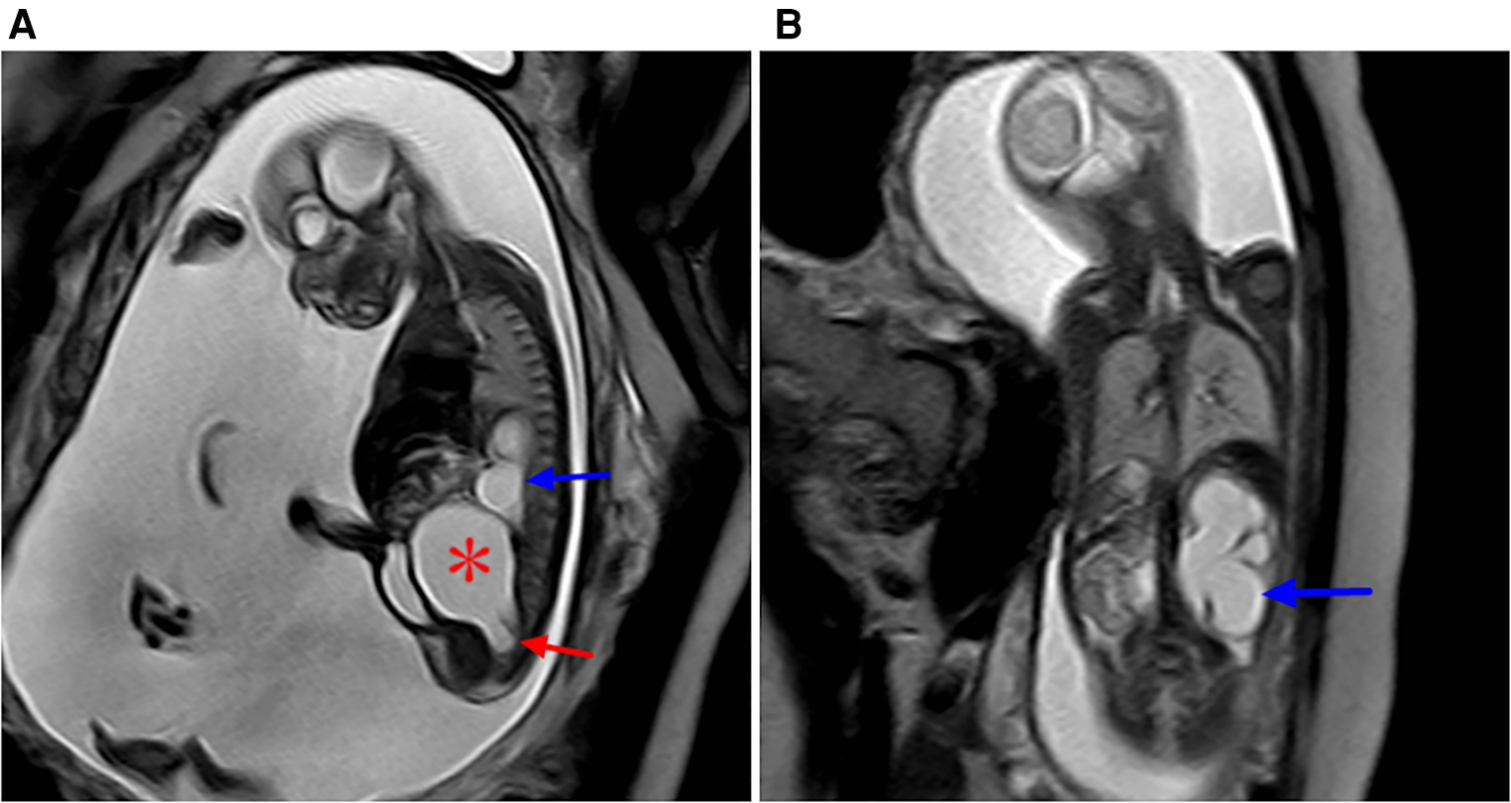
Sacrococcygeal teratoma type IV (misdiagnosed as dilated ureter by prenatal ultrasound) at 24 weeks of gestation. (**A**) Sagittal view of the sacral coccyx shows a large cystic lesion in the anterior sacral pelvic cavity (red star) that is closely related to the coccyx below (red arrow), with a dilated left hydronephrosis (blue arrow) and increased amniotic fluid. (**B**) Coronal view of the abdomen and hydronephrosis of the left kidney (blue arrow).

Among the 60 pregnant women, 59 had a singleton pregnancy, while one had a twin pregnancy. The specific MRI diagnosis results for the fetal abdominal cavity or sacrococcygeal masses were as follows:

(1) Fifty-three cases of sacrococcygeal masses were diagnosed by prenatal MRI, including teratoma in 52 cases and a parasitic fetus in one case (Figure 1). MRI-based diagnoses of sacrococcygeal teratomas categorized 21 cases in type I (Figure 2), 20 cases in type II (Figure 3), eight cases in type III, and three cases in type IV (Figure 4). MRI of sacrococcygeal teratoma usually showed cystic or confounding signal masses, which were closely related to the caudal vertebra, but there was no communication between the lesions and the spinal lumen and the position of the conus spinal cord was normal. In type II or III lesions, the rectum was compressed forward, and T1WI sequences of the fetal or neonatal period showed that the rectum was in front of the lesion with a high signal (Figures 3D, 4B). Sacrococcygeal teratoma may cause ureteral compression, resulting in hydronephrosis (Figure 4C), secondary fetal edema, and peritoneal effusion (Figure 4D), and severe hydronephrosis may lead to renal parenchymal necrosis and calcification (Figure 4E). In our study, 22 cases of sacrococcygeal teratomas were complicated with hydramnios, five involved placental edema, and three involved fetal hydronephrosis (all type IV; see Table 1 for details).
(2) In seven cases, the parasitic fetuses were located in the left upper abdomen. The parasitic fetus was characterized as a well-defined mass with cystic components, in which the skeleton and spinal structure of the embryo were visible (Figures 5, 6). As the pregnancy continued, the parasitic fetus may gradually increase in size (Figures 5C–E, 6B, C). No four-chamber heart structure was found in the parasitic fetus, but spinal structure was found in three cases and limb structure in seven cases. More details are provided in Table 2.
(3) MRI misdiagnosed one case of type IV sacrococcygeal teratoma as a cloacal malformation (Figure 7). The fetus presented as a pelvic cystic mass, which was divided into left and right halves with a visible internal separation (Figure 7A). Fetal MRI misdiagnosed it as a double uterine malformation accompanied by bilateral hydronephrosis (Figure 7B), and cystic lesions with multiple compartments were observed in postnatal assessments. Subsequently, sacrococcygeal teratoma originating in the coccyx was diagnosed (Figure 7C). Prenatal ultrasound misdiagnosed one case of type IV sacrococcygeal teratoma complicated with hydronephrosis as a dilated ureter. Prenatal MRI showed anterior sacrococcygeal cystic lesions closely related to the sacrococcygeal bone (Figure 8A), bladder compression forward, and a dilated left hydronephrosis (Figure 8B).

**Table 1:** Prenatal and postnatal follow-up findings in 52 cases of fetal sacrococcygeal teratoma.

Type	MA (years)	Sex		GA (weeks)	Complications			Progress	
		Male	Female		Hydramnios	Placenta edema	Hydronephrosis	ND	IL
Ⅰ	27.67 ± 5.03	6	15	25.48 ± 3.77	9	0	0	18	2
Ⅱ	28.00 ± 5.95	4	16	27.08 ± 3.81	11	3	0	17	3
Ⅲ	28.12 ± 5.66	0	8	28.73 ± 5.30	1	1	0	6	1
Ⅳ	25.75 ± 4.34	1	2	31.00 ± 4.83	1	1	3	2	1

MA, maternal age; GA, gestational age; ND, normal delivery; IL, induced labor.

**Table 2:** Prenatal data and postnatal follow-up findings in 8 cases of fetal parasitic fetuses.

Location	MA (years)	Sex		GA (weeks)	Progress	
		Male	Female		ND	IL
Abdominal	26.43 ± 1.9	7	0	28.88 ± 4.47	6	1
Sacrococcygeal	27	0	1	25 + 2 days	1	0

MA, maternal age; GA, gestational age; ND, normal delivery; IL, induced labor.

### 3.2. Comparison of prenatal MRI with prenatal ultrasound and follow-up assessments

Sixty cases of abdominal or caudal masses. The 52 teratomas were observed in 11 male and 41 female fetuses, while the parasitic fetuses included one female (caudal) and seven male (all located in the abdominal cavity) fetuses.

(1) Follow-up results: Fetal induction of labor was observed in eight cases (8/60), including one case of parasitic fetus and seven cases of sacrococcygeal teratomas. Pregnancy was continued in 52 cases, including 7 cases with parasitic fetuses and 45 with teratomas. Among these, 51 cases involved postnatal operations and showed normal growth and development in follow-up assessments, only one case of fetal type IV sacrococcygeal teratoma was complicated with severe hydronephrosis at 32 weeks of gestation, and treatment was abandoned due to blood accumulation in both kidneys, hydrops abdominis, and edema in both lower limbs after birth.
(2) Comparison of fetal MRI and follow-up results: The prenatal MRI diagnosis and follow-up diagnosis were identical in 51 cases, indicating an accuracy of 98.08% (51/52). One case of sacrococcygeal teratoma was misdiagnosed as a cloacal malformation by prenatal MRI (Figure 7). All eight cases of parasitic fetuses were diagnosed correctly by fetal MRI.
(3) Comparison of prenatal ultrasound and follow-up results: Among the 52 cases of fetal teratomas, 39 were correctly diagnosed by prenatal ultrasound. Among the remaining 13 cases, no definite diagnosis was obtained and only intraperitoneal mixed echo or presacral mass was suggested in seven cases (Figure 6A), while six cases were misdiagnosed as showing a dilated ureter (2 cases), spina bifida with meningocele (2 cases), cloacal malformation (1 case), or meconium peritonitis (1 case; Figure 4A). Five cases of parasitic fetuses were misdiagnosed as teratomas (3 cases), adrenal cyst (1 case), or pancreatic cystadenoma (1 case).

## 4. Discussion

### 4.1. Clinical and MRI features of abdominal and sacrococcygeal teratomas or parasitic fetuses

The fetal sacrococcygeal region originates from the progenitor node or Hensen's node of the embryonic progenitor strip. When the progenitor node persists due to developmental disorders, an teratoma is formed in the sacrococcygeal region (7). Sacrococcygeal teratoma occurs in approximately 1:35,000–40,000 births and affects approximately 1 in 27,000 pregnancies (11). Approximately 75% of affected infants are female (12). In our study, female patients accounted for 79.2% of the patients with sacrococcygeal teratomas.

The most common complications in the present study were an increased fetal heart-to-chest ratio, fetal edema, and excessive amniotic fluid, and considering the risk of maternal mirror syndrome and ultimate fetal demise, the mortality rate may be as high as 50% (1, 13, 14). The goal at MR imaging is to characterize the tumor morphology and possible extensions into the spinal canal, and provide evidence of mass effect manifested by bowel/bladder obstruction that may progress to hydronephrosis (15). This study showed that the probability of sacrocaudal teratoma complicated with polyhydramnios was 41.5%, while the corresponding value for placenta edema was 11.3%. However, none of the cases showed fetal heart death because of high cardiac output. Only one case of type IV sacrococcygeal teratoma was found to be associated with severe hydrops abdominis and edema in both lower limbs after birth, which may be attributed to compression of the kidney with hydronephrosis and renal necrosis, Thus, for type IV sacrococcygeal teratoma, closer follow-up is required, and severe hydronephrosis may require early interventions such as intrauterine surgery to avoid secondary fetal edema and severe renal and parenchymal necrosis. Fetuses with hydrops that underwent a fetal intervention had a survival rate of 38%, in comparison with the survival rate of 9% for fetuses with no intervention (16). Infants with solid tumors are at a higher risk of developing high-output cardiac failure and hydrops, which has an overall worse prognosis (17). In our study, one teratoma with complications after birth showed solid components, while other teratomas with cystic or solid components all showed good condition after the operation. The overall prognosis of type IV sacrococcygeal solid teratomas may be poor, which is consistent with the findings of previous reports (13).

A parasitic fetus is relatively rare, with the retroperitoneum being the most common site and other reported sites including the head, sacrum, scrotum, mediastinum, lung, and kidneys (18). However, the most common site of the parasitic fetus in our study was the abdominal cavity (7/8 cases, 87.5%). Parasitic fetuses also show a 2:1 male predominance, unlike the female predominance of teratomas (19), and the parasitic fetuses in our study also showed a male predominance, with all seven cases of abdominal cavity parasitism being male and only one case of sacrococcygeal parasitism being female. Prenatal diagnosis of these fetuses is relatively easy, and mainly involves identification of the structure of the spine or limbs. On MRI, parasitic fetuses often present as a cyst with clear boundaries, and the cyst is mainly composed of liquid components, with SSFSE showing a high signal and the fetal limbs or spine in the cyst presenting an equal or low signal. The fetal parasitic fetus can contain blood supply vessels, and will continue to increase with fetal growth, but the capsule is clear, so it mainly changes the surrounding oppression, generally does not invade the surrounding tissue, and can be completely cured by resection. The well-differentiated structures (with or without vertebral column) should be enclosed within a distinct sac and should connect to the host twin by a few vessels located near the attachment site, instead of showing a broad attachment with multiple feeding vessels (20, 21).

### 4.2. Differential diagnosis

Sacrococcygeal teratomas usually need to be distinguished from myelomeningocele or meningocele as well as cloacal malformations. (1) Sacrococcygeal meningomyelocele can be located in the front or rear of the sacrococcygeal region, mostly in the rear, and contain cerebrospinal fluid cystic components, but the lesions are connected with the spinal lumen, we can accurately determine the relationship between the lesions and the spinal lumen on the cross-sectional and sagittal plane images. Although teratomas have been reported to extend into the spinal lumen (22), the overall probability of such extension is low, and our study included no case of teratoma communicating with the spinal lumen. (2) Cloacal malformation is rare and occurs in female fetuses, The incidence of cloacal malformations has been reported to be 1 in 50,000 to 1 in 250,000 (23) and these malformations typically present as a repeated uterus and vagina with cystic dilatation, which needs to be distinguished from sacrococcygeal teratoma. Compression of the ureter, which results in dilation of the ureter or hydronephrosis, is more common in cloacal malformations than in sacrococcygeal teratoma. The position of the rectum on the T1WI sequence is helpful to differentiate between cloacal malformations and sacrococcygeal teratomas, with the former showing the rectal lumen with a high signal behind the cystic lesion (dilated uterus vagina) and the latter showing the rectum in front of the lesion.

Parasitic fetuses should be distinguished from teratomas. Parasitic fetuses are bordered by a clear water sac, with the lesions containing limb bones or a spinal structure and showing equal or low signal on MRI, while teratomas usually show a mixed signal with no limbs or spinal structures. Moreover, parasitic fetuses contain blood supply and can also continue to increase with the growth of the fetus. The parasitic fetuses in our group all increased after birth. Another interesting finding in this study was that the location of fetal parasitic fetuses was quite characteristic [left upper abdomen of the fetus in 7/8 cases (87.5%)], and all fetuses with abdominal parasitic fetuses were male; thus, the location of the disease should also serve as a useful identification point.

The different diagnostic accuracy of prenatal ultrasound in comparison with MRI can be attributed to the following reasons: (1) The diagnosis in prenatal MRI was obtained on the basis of the prenatal ultrasound diagnosis. Thus, if the results of prenatal ultrasound are not known in advance, the diagnostic positive rate or accuracy of MRI may decrease. (2) When displaying fetal abdominal and pelvic lesions, MRI can reveal the findings for arbitrary sections or show multi-sequence imaging, allowing more accurate identification of lesion location than prenatal ultrasound. Thus, accurate positioning plays a crucial role in qualitative diagnosis of the lesions.

### 4.3. Perinatal management and prognosis of fetal teratoma or parasitic fetus

When prenatal ultrasound shows abdominal or sacrococcygeal masses, MRI can be recommended for further examination. In addition to providing diagnostic information, MRI can also serve as an important guide to treatment, delivery planning, and counseling. MRI provides a large field of vision, high-resolution soft-tissue imaging, and arbitrary section imaging, and is not easily affected by fetal position in the third trimester, which plays a role in the localization and qualitative diagnosis of lesions. Our study also showed that MRI can yield more information than ultrasound.

Fetal teratomas or parasitic fetuses usually have a good prognosis after surgery. The prognosis of fetal sacrococcygeal teratomas depends on the location and size of the tumor and any associated perinatal complications (4). Fetal edema and teratoma have been reported to contain solid components, and a sacrococcygeal mass to fetal weight ratio greater than 0.12 at 24 weeks of pregnancy may be a risk factor (17). Prenatal counseling is often required after prenatal ultrasound, with the prospective parents receiving an explanation from MRI experts or a professional consultation with a neonatal surgeon to fully understand the disease prognosis, and it can reduce the rate of induced labor. For early fetal complications with severe hydronephrosis, close follow-up or prompt intervention can reduce the risk of poor prognosis of renal necrosis and fetal edema after birth.

## Data Availability

The original contributions presented in the study are included in the article/Supplementary Material, further inquiries can be directed to the corresponding author.
